# Dupilumab for the treatment of COPD: protocol for a systematic review and meta-analysis

**DOI:** 10.3389/fmed.2025.1636238

**Published:** 2025-07-21

**Authors:** Bingyu Xue, Qionghua Xiao, Yuanming Huang, Minghang Wang

**Affiliations:** ^1^National Regional Medical Center of Traditional Chinese Medicine (TCM) (Pulmonary Disease), The First Affiliated Hospital of Henan University of Chinese Medicine, Zhengzhou, Henan, China; ^2^The First Clinical Medical School, Henan University of Chinese Medicine, Zhengzhou, Henan, China

**Keywords:** dupilumab, COPD, systematic review, meta-analysis, type 2 inflammation

## Abstract

**Introduction:**

Chronic obstructive pulmonary disease (COPD) is a significant global public health issue, with a high incidence and mortality burdening patients heavily. Although existing treatment methods have made certain progress, their effectiveness in intervention during acute exacerbations and long-term management remains suboptimal for some patients. In recent years, biologic agents have provided new directions for COPD treatment. Dupilumab, a humanized monoclonal antibody targeting IL-4Rα, has shown good efficacy in type 2 inflammation-related diseases; however, its efficacy and safety in COPD treatment remain to be verified.

**Methods and analysis:**

This study will assess the efficacy and safety of dupilumab in the treatment of COPD through a systematic review and meta-analysis. Randomized controlled trials (RCTs) meeting the inclusion criteria will be included, and the Population-Intervention-Comparator-Outcome-Study (PICOS) research design standard will be adopted to compare the efficacy of dupilumab against placebo or conventional treatment. The primary outcome will be the annual exacerbation rate (AER) of COPD, and the secondary outcomes will include changes in FEV1 before baseline bronchodilators, St. George’s Respiratory Questionnaire (SGRQ) scores, Evaluating Respiratory Symptoms in COPD (E-RS COPD) and blood eosinophil counts. Safety will also be assessed, and the incidence of adverse reactions will be statistically analyzed. A literature search will be conducted across multiple databases, including PubMed, Embase, WOS, Cochrane Library, and Scopus. Ultimately, this study will assess the impact of dupilumab on COPD patients through meta-analysis, aiming to provide new evidence-based insights for the treatment of COPD.

**Ethics and dissemination:**

Ethical approval is not required for this review. Upon completion, the results of this review will be submitted to peer-reviewed journals for publication and/or presented at academic conferences.

**Trial registration number:**

https://www.crd.york.ac.uk/prospero/, identifier CRD42024605417.

## Introduction

Chronic obstructive pulmonary disease (COPD) is a chronic respiratory condition characterized by persistent respiratory symptoms and irreversible airflow limitation. Its core pathology involves abnormal inflammatory responses of the airways and lung tissue to harmful stimuli (1). Currently, COPD has risen to become the third leading cause of death worldwide. Epidemiological data indicate that, in 2021, the number of deaths attributed to COPD globally reached 3.5 million, accounting for approximately 5% of all-cause deaths (2). It is projected that, by 2050, the global number of COPD patients will exceed 600 million (3). Although current mainstream treatment options (such as bronchodilators combined with glucocorticoids) can alleviate certain clinical symptoms, they still face two major challenges: firstly, some patients still experience frequent acute exacerbations and continuous decline in lung function, which has become a bottleneck in treatment; secondly, long-term use of inhaled glucocorticoids (ICS) may induce adverse reactions such as pneumonia and osteoporosis, and their therapeutic effects still exhibit significant limitations (4).

It is noteworthy that recent studies have identified 20%–40% of COPD patients as exhibiting Th2 inflammatory phenotypic characteristics (5). These patients show poor response to traditional anti-inflammatory treatments but may benefit from precise interventions targeting the Th2 pathway. Dupilumab, a fully humanized monoclonal antibody, can simultaneously inhibit IL-4 and IL-13 signal transduction by specifically blocking the IL-4 receptor alpha subunit (IL-4Rα), thereby effectively regulating Th2-type inflammatory responses. This drug has demonstrated outstanding efficacy in type 2 inflammation-related diseases (such as moderate to severe asthma) (6), but its value in the treatment of COPD still requires evidence-based verification. Although recent findings from the phase III clinical trials BOREAS and NOTUS (7, 8), along with related cohort study (9), offer initial evidence supporting dupilumab’s use in COPD, the available studies differed in their results in terms of efficacy metrics and safety outcomes. In addition, the sample sizes of individual studies are relatively small, and the statistical efficacy of a single trial is insufficient to fully explain the clinical heterogeneity. Therefore, a meta-analysis system integrates evidence from multiple sources, quantitatively assesses the combined effects of efficacy and safety, and explores sources of heterogeneity, with the aim of providing valuable evidence for clinical decision-making and future research.

## Methods

### Study registration

This protocol has been formally registered on the PROSPERO platform (registration number: CRD42024605417), with the initial registration date of 29 October 2024. The research report will strictly adhere to the Preferred Reporting Items for Systematic Reviews and Meta-Analyses Protocol (PRISMA-P) 2015 statement (10). The detailed PRISMA-P 2015 checklist is attached as Supplementary File 1. The research will officially commence on 1 December 2024, with the completion of all tasks expected by 30 August 2025.

### Inclusion criteria

The Population-Intervention-Comparator-Outcome-Study (PICOS) research design framework will be used as the basis for determining the inclusion criteria of the primary studies.

### Population

The participants included in this study are adult patients diagnosed with COPD according to the 2025 Global Initiative for Chronic Obstructive Lung Disease (GOLD) guidelines (11), with no additional restrictions based on gender, ethnicity, or other demographic characteristics.

### Intervention

The intervention includes the treatment regimen with dupilumab, with no limitations on dosage or duration.

### Comparison

The control group interventions are placebos or conventional medical treatments (e.g., bronchodilators, inhaled glucocorticoids, etc.).

### Outcomes

The studies reporting one or more of the following outcomes will be included.

#### Primary outcomes

Annualized rate of exacerbations (AER) of COPD.

#### Secondary outcomes

The change from baseline in prebronchodilator FEV1, St. George’s Respiratory Questionnaire (SGRQ), Evaluating Respiratory Symptoms in COPD (E-RS COPD), and blood eosinophil counts will be measured. Additionally, information will be collected on adverse events and serious adverse events associated with dupilumab to evaluate its safety in patients with COPD.

### Study design

The study design incorporates both randomized controlled trials (RCTs) and cohort studies. We explicitly plan to include pivotal clinical trials that provide direct evidence of the efficacy of dupilumab in the treatment of type 2 inflammatory COPD, in particular the BOREAS trial, the NOTUS trial, and the related cohort study, whose results will be critical to evaluating the efficacy and safety of dupilumab in the target population.

### Exclusion criteria

The following types of studies will be excluded: case series, case reports, and cross-sectional study; studies for which the full text cannot be obtained for various reasons; studies with duplicate, missing, or non-extractable data (e.g., numerical results were not reported, data were presented only graphically, etc.); studies unrelated to the topic of this research; and non-English literature.

### Information sources and search strategy

The relevant literature will be searched in the PubMed, Web of Science, Embase, Cochrane, and Scopus databases, with the search span covering the period from the establishment of each database to 31 March 2025. The search terms will include “Dupilumab,” “COPD,” and others. The search strategy used in the PubMed database is provided in Table 1, and the complete search strategy is included in Supplementary File 2. The literature search will be conducted independently by two researchers (BX and QX). In case of disagreement, a third researcher (MW) will participate in the discussion to reach a consensus.

**TABLE 1:** Search strategy in PubMed.

No.	Search terms
#1	Chronic Obstructive Pulmonary Diseases [Mesh terms] or COPD or Chronic Obstructive Lung Disease or Chronic Obstructive Pulmonary Disease or COAD or Chronic Obstructive Airway Disease or Airflow Obstruction, Chronic or Airflow Obstructions, Chronic or Chronic Airflow Obstructions or Chronic Airflow Obstruction
#2	Dupilumab or Dupixent or SAR231893 or SAR-231893 or REGN668 or REGN-668
#3	#1 AND #2

### Study selection and data collection

#### Study selection

**FIGURE 1:**
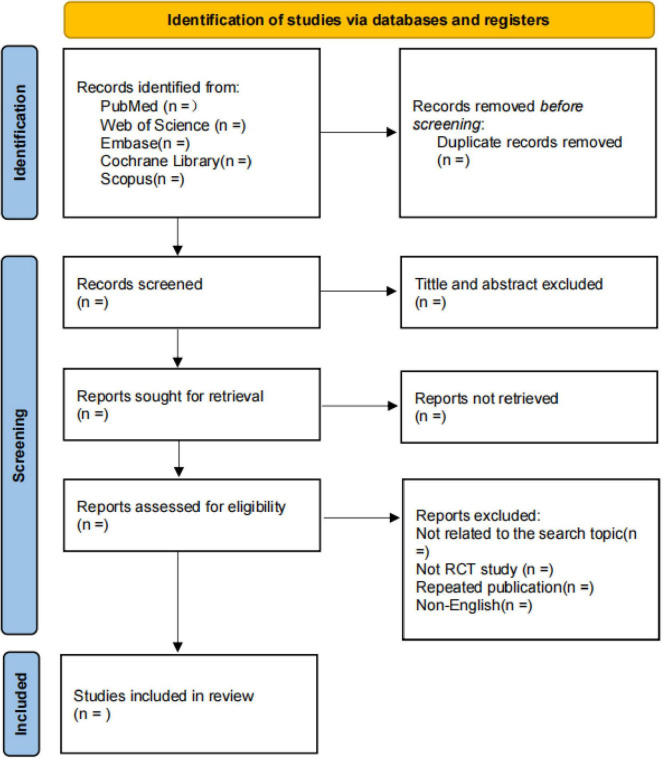
Flowchart of literature screening process.

Two researchers (BX and YH) will independently read and screen the literature based on the established inclusion and exclusion criteria, extract the required data, and cross-check the data. The literature screening process is as follows: two researchers will use NoteExpress 3.0 software to remove duplicate or concurrently published articles across databases. They will independently review the titles and abstracts of the articles, exclude those unrelated to the study, and then perform full-text reading on the remaining studies that may meet the inclusion criteria. Studies with consistent agreement will be included; in case of disagreement, a third researcher (MW) will assist in resolving the issue. The detailed flowchart is provided in Figure 1.

#### Data extraction

Data extraction will be conducted independently by two researchers (BX and YH). The data will be organized using Excel 2019, and the following information will be extracted:

1. Participants: male gender ratio, age, smoking history, FeNO, and eosinophil count.
2. Interventions: dupilumab dosage and treatment duration.
3. Comparators: type of comparator.
4. Outcomes: the primary outcome is the annualized rate of exacerbations (AER) of COPD. Additionally, at each measurement time point, the following data will be recorded: pre-bronchodilator FEV1, SGRQ, and E-RS COPD. For continuous variables (including pre-bronchodilator FEV1, SGRQ scores, and E-RS COPD scores), effect sizes will be expressed using mean difference (MD) or standardized mean difference (SMD). The type of effect size, effect size for efficacy and safety outcomes, and the incidence of adverse events and serious adverse events will also be recorded.
5. Study design: study type, sample size, heterogeneity, and publication bias.
6. Other: first author of the study, year of publication.

In case of discrepancies, a third researcher (MW) will assist in resolving the issue if necessary.

### Risk of bias assessment

For RCTs, the methodological quality of the results from the randomized trials will be assessed by two independent reviewers (QX and YH) using the revised Cochrane risk of bias assessment tool (12). This tool covers the following five areas: (1) the randomization process; (2) deviations from intended interventions; (3) missing outcome data; (4) measurement of the outcome; and (5) selection of the reported result. The assessment process will adopt a three-level judgment system: each item will be independently rated as “low risk” (compliant with standards), “high risk” (indicating systematic bias), or “concerns” (partially non-compliant). In case of disagreement between the reviewers, resolution will be sought through consultation or by involving a third reviewer (MW) to mediate until consensus is reached.

For cohort studies, risk of bias assessment will be evaluated with the Newcastle-Ottawa Scale (13). The Newcastle-Ottawa Scale mainly assesses the risk of bias in three core areas: selection (focusing on the representativeness and determination of the exposed and non-exposed groups), comparability (focusing on the control of important confounding factors) between groups, and outcome (focusing on the independence, objectivity of outcome assessment and completeness of follow-up), to comprehensively evaluate the methodological quality of the studies. If there are differences among the reviewers, consensus will still be reached through consultation or by the intervention and mediation of a third reviewer (MW).

### Statistical analysis

This study will conduct statistical analysis using the “meta” package in R 4.3.3 software, with effect sizes presented as MD, SMD, or risk ratio (RR) and their 95% confidence intervals (CIs); for continuous outcome measures (e.g., change in FEV1), MD will be used if the studies used the same unit of measurement and scale, and SMD will be used to standardize the results and enable combinations if the studies used different units of measurement or scales. Firstly, heterogeneity between studies will be assessed using the Cochran *Q* test and *I*^2^ statistic. If the *P*-value is ≥0.1 and *I*^2^ ≤ 50%, a fixed-effect model will be used; if the *P*-value is <0.1 or *I^2^* > 50%, significant heterogeneity will be indicated, and a random-effects model will be applied (14). Next, the combined MD/SMD/RR and their 95% CI for each outcome measure will be calculated, and the results will be presented in text, tables, and graphs. The statistical significance level is set at *P* < 0.05.

### Subgroup analysis

Subgroup analyses will be conducted when heterogeneity is detected and sufficient data are available, with the aim of further comparing differences in primary and secondary outcomes across various factors. The main factors for analysis include: demographic characteristics (age, gender, race/ethnicity, and body mass index), biomarkers (blood eosinophil, count, with a threshold set at ≥300 cells/μl, and FeNO, with the threshold set at ≥20 ppb), disease characteristics (baseline disease severity, history of previous acute exacerbations, and presence of emphysema), smoking status (current or former smoker), treatment background (inhaled corticosteroid dosage, triple therapy, etc.), study design type (RCT, cohort study) and geographic region (country or regional stratification).

### Assessment of publication bias

The assessment of publication bias will be conducted through funnel plots and the Egger intercept test. If the funnel plot is asymmetrical, it will indicate a higher likelihood of publication bias. The Egger test will be used to further confirm the bias. The *P*-value of <0.05 will be considered evidence of publication bias, while a *P*-value of ≥0.05 will indicate the absence of bias (15, 16).

### Sensitivity analysis

Sensitivity analysis will include both influence analysis and meta-analysis: influence analysis will involve sequentially excluding each study to qualitatively assess its impact on the final outcome; meta-analysis will involve excluding trials with overall higher risk and conducting quantitative analysis to assess the robustness of the results (17).

## Discussion

Chronic obstructive pulmonary disease is one of the major global health issues. Despite some progress in current treatment methods, the high incidence and mortality of COPD continue to impose a significant health burden on patients (18–20). In recent years, biologic agents have gradually emerged as a significant treatment option for COPD, particularly in patients with type 2 inflammation-associated COPD. Dupilumab, a humanized monoclonal antibody targeting IL-4Rα, can significantly modulate Th2-type inflammatory responses and reduce airway inflammation by inhibiting the IL-4 and IL-13 signaling pathways. For COPD patients with significant Th2-type inflammation, dupilumab offers a new therapeutic option, and its therapeutic effects has been validated in the treatment of other type 2 inflammation-associated diseases (21–23). However, the efficacy and safety of dupilumab in the treatment of COPD still require further verification through systematic review and meta-analysis.

This study employed systematic review and meta-analysis to comprehensively evaluate the efficacy and safety of dupilumab in the treatment of COPD. The results of this study not only provide new therapeutic directions for COPD patients but also offer important decision-making guidance for clinicians. Particularly, in the context of limited existing treatment options, the introduction of dupilumab may bring significant clinical benefits to a subset of patients. Through this study, both academia and clinical practice can gain deeper insights into the potential application of dupilumab in COPD patients, providing guidance for future research and clinical treatment.

This study demonstrates significant methodological distinctions from existing descriptive systematic reviews and does not merely replicate prior research. For instance, while Freund et al.’s (24) investigation focused on real-world applications of biologics and patient-reported outcomes, it was limited to descriptive synthesis without employing quantitative methods to assess overall effect sizes. Furthermore, the real-world evidence incorporated primarily comprised retrospective case reports, which were considered to have relatively limited evidentiary value. On the other hand, a quantitative analysis of U.S. healthcare claims data revealed that dupilumab treatment for COPD was associated with significantly fewer adverse events compared with ICS/LABA therapy, providing supporting evidence for its long-term safety profile (9).

This study does not simply replicate existing research but strictly follows the PRISMA-P guidelines, including high-quality RCTs and cohort studies that meet the PICOS framework (25), and quantifies the comprehensive effect of dupilumab on core outcome indicators in COPD treatment through meta-analysis. This approach not only overcomes the limitations of previous descriptive reviews but also explores the potential impact of patient characteristics on efficacy through subgroup analysis, providing stratified evidence for precision medicine. Additionally, this study further ensures the reliability of the results through sensitivity analysis and publication bias assessment, making up for the methodological rigor deficiencies in existing studies and providing a more scientific basis for the application of dupilumab in COPD treatment.

In addition, several limitations remain in this study. Firstly, the inclusion of only English-language publications may have led to the omission of relevant studies published in other languages, thereby affecting the comprehensiveness of the evidence. Secondly, research on the use of dupilumab in the treatment of COPD is still in its early stages, and the publicly available data are relatively limited, which may increase the risk of selection bias and publication bias.

## Study period

This systematic review will be conducted from 1 December 2024 to 30 August 2025. Of this, the literature search will be completed by 31 March 2025 to ensure sufficient time for subsequent data extraction, quality evaluation, etc.
