# Supplementary material for: Dupilumab for the treatment of COPD: protocol for a systematic review and meta-analysis
Source: Front Med (Lausanne). 2025 Jul 21;12:1636238. doi: 10.3389/fmed.2025.1636238 (PMC12318725; doi:10.3389/fmed.2025.1636238)
Supplement: Supplementary file 2 [file Supplementary_file_2.docx]

**Supplementary file 2.** The details of the search strategy.

| **Pubmed** |
| --- |
| ((Chronic Obstructive Pulmonary Diseases or COPD or Chronic Obstructive Lung Disease or Chronic Obstructive Pulmonary Disease or COAD or Chronic Obstructive Airway Disease or Airflow Obstruction, Chronic or Airflow Obstructions, Chronic or Chronic Airflow Obstructions or Chronic Airflow Obstruction)) AND (Dupilumab or Dupixent or SAR231893 or SAR-231893 or REGN668 or REGN-668)  (((((((((Chronic Obstructive Pulmonary Diseases[MeSH Terms]) OR (COPD[Title/Abstract])) OR (Chronic Obstructive Lung Disease[Title/Abstract])) OR (Chronic Obstructive Pulmonary Disease[Title/Abstract])) OR (COAD[Title/Abstract])) OR (Chronic Obstructive Airway Disease[Title/Abstract])) OR (Airflow Obstruction, Chronic[Title/Abstract])) OR (Airflow Obstructions, Chronic[Title/Abstract])) OR (Chronic Airflow Obstructions[Title/Abstract])) OR (Chronic Airflow Obstruction[Title/Abstract])  (((((Dupilumab[MeSH Terms]) OR (Dupixent[Title/Abstract])) OR (SAR231893[Title/Abstract])) OR (SAR-231893[Title/Abstract])) OR (REGN668[Title/Abstract])) OR (REGN-668[Title/Abstract]) |
| **Embase** |
| #1 **'dupilumab'**/exp OR **'dupilumab'** OR **'dupixent'**/exp OR **'dupixent'**  #2**'chronic obstructive pulmonary diseases'** OR **'copd'**/exp OR **'copd'** OR **'chronic obstructive lung disease'**/exp OR **'chronic obstructive lung disease'** OR **'chronic obstructive pulmonary disease'**/exp OR **'chronic obstructive pulmonary disease'** OR **'coad'** OR **'chronic obstructive airway disease'** OR **'airflow obstruction, chronic'** OR **'airflow obstructions, chronic'** OR **'chronic airflow obstructions'** OR **'chronic airflow obstruction'**/exp OR **'chronic airflow obstruction'**  #1 AND #2 |
| **Cochrane Library** |
| #1 MeSH descriptor: [Pulmonary Disease, Chronic Obstructive] explode all trees  #2 (COPD):ti,ab,kw or (Chronic Obstructive Lung Disease):ti,ab,kw or (Chronic Obstructive Pulmonary Disease):ti,ab,kw or (COAD):ti,ab,kw or (Chronic Obstructive Airway Disease):ti,ab,kw or (Airflow Obstruction, Chronic):ti,ab,kw or (Airflow Obstructions, Chronic):ti,ab,kw or (Chronic Airflow Obstructions):ti,ab,kw or (Chronic Airflow Obstruction):ti,ab,kw  #3 #1 OR #2  #4 Dupilumab  #5 Dupixent  #6 #4 OR #5  #7 #3 OR #6 |
| **Web of Science** |
| (TS=(Chronic Obstructive Pulmonary Diseases or COPD or Chronic Obstructive Lung Disease or Chronic Obstructive Pulmonary Disease or COAD or Chronic Obstructive Airway Disease or Airflow Obstruction, Chronic or Airflow Obstructions, Chronic or Chronic Airflow Obstructions or Chronic Airflow Obstruction)) AND TS=(Dupilumab or Dupixent or SAR231893 or SAR-231893 or REGN668 or REGN-668) |
| **Scopus** |
| ( TITLE-ABS-KEY ( chronic AND obstructive AND pulmonary AND diseases ) OR TITLE-ABS-KEY ( copd ) OR TITLE-ABS-KEY ( chronic AND obstructive AND lung AND disease ) OR TITLE-ABS-KEY ( chronic AND obstructive AND pulmonary AND disease ) OR TITLE-ABS-KEY ( coad ) OR TITLE-ABS-KEY ( chronic AND obstructive AND airway AND disease ) OR TITLE-ABS-KEY ( airflow AND obstruction, AND chronic ) OR TITLE-ABS-KEY ( airflow AND obstructions, AND chronic ) OR TITLE-ABS-KEY ( chronic AND airflow AND obstructions ) OR TITLE-ABS-KEY ( chronic AND airflow AND obstruction ) ) AND ( TITLE-ABS-KEY ( dupilumab ) OR TITLE-ABS-KEY ( dupixent ) ) |
